# Medical Items

**Published:** 1894-02

**Authors:** 


### MEDICAL ITEMS.

Dr. J. Kennedy Corss, late of Philadelpia, has located in Atlanta.

Dr. Frank H. Sims has resigned the chair of Eye, Ear, Nose and Throat in the Atlanta Polyclinic.

M. Brissaud will succeed the late Dr. Charcot at the Salpetrière, Paris, until a permanent successor is appointed.

Dr. E. Burke Haywood of Raleigh, N. C., died January 18. Dr. Haywood was one of che ablest and most prominent physicians in North Carolina, and died full of years and honors.

Dr. W. C. Jarnagin and Miss Erskine Richmond, both of Atlanta, were married on the 17th ultimo. The Journal congratulates both parties and wishes them a long life of happiness.

Dr. Abraham Jacobi was recently offered the chair of Diseases of Children in the University of Berlin. Dr. Jacobi promptly declined the invitation, preferring to remain in New York.

Dr. J. H. Blue, of Montgomery, Ala., committed suicide January 8th on account of compromising relations with a female patient. He was forty-four years of age, and a physician of large practice

The following officers have been elected by the Atlanta Society of Medicine for the year 1894: President, Dr. J. W. Duncan; Vice-President, Dr. C. D. Hurt; Secretary, Dr. R. R. Kime; Treasurer, Dr. L. B. Grandy; Corresponding Secretary and Librarian, Dr. N. O. Harris.

We have received the first issue of the *Atlanta Polyclinic*, published monthly by the faculty of the Atlanta Polyclinic, and edited by Dr. J. Arthur Childs. The issue contains some good articles written by members of the faculty, and also some well selected items and news of medical interest. We take pleasure in offering the *Polyclinic* and the Polyclinic our very best wishes.

At a meeting of the faculty of Jefferson Medical College, held January 8th, 1894, it was unanimously resolved to institute a compulsory four years' course with the session of 1895–'96. This step was taken in order that the large clinical service of the Jefferson College Hospital (350 cases a day) might be utilized to the fullest extent in carrying out the desire of the faculty to provide advanced medical education of a practical character.

The South Georgia Medical Association has been permanently organized at Helena, Ga., and will meet quarterly on the first Tuesdays in March, June, September and December. It has a membership of about forty regular physicians, with Dr. G. W. Blanton, of Chauncey, President, and Dr. H. J. Smith, of McRae, and J. D. Herman, of Eastman, Vice-Presidents, and Joseph A. Estes, of Eastman, Secretary and Treasurer. This Association is intended as an ally to the Georgia Medical Association. All the physicians in the State who are graduates of a regular school of medicine in good standing are invited to membership.

---

AN Army Medical Board will be in session at Washington City, D. C., during April, 1894, for the examination of candidates for appointment to the Medical Corps of the United States Army, to fill existing vacancies.

Persons desiring to present themselves for examination by the Board will make application to the Secretary of War before March 15, 1894, for the necessary invitation, giving the date and place of birth, the place and State of permanent residence, the fact of American citizenship, the name of the medical college from which they were graduated, and a record of service in hospital, if any, from the authorities thereof. The application should be accompanied by certificates, based on personal acquaintance, from at least two reputable persons, as to his citizenship, character and habits. The candidate must be between twenty-two aud twenty-eight years of age, and a graduate from a regular medical college, as evidence of which his diploma must be submitted to the Board.

Successful candidates at the coming examination will be given a course of instruction at the next session of the Army Medical School, beginning in November, 1894.

Further information regarding the examination may be obtained by addressing the Surgeon-General United States Army, Washington, D. C. GEO. M. STERNBERG,

Surgeon-General U. S. Army.

---

MRS. H. V. M. MILLER, wife of Dr. Miller, died January 8th. Following are resolutions passed by students of Atlanta Medical College:

ATLANTA, GA., January 9th, 1894.

WHEREAS, In the exercise of His loving Providence, the Omniscient God has seen fit to take to himself the estimable wife of Dr. H. V. M. Miller, thus severing that sacred bond of companionship enjoyed by them during so many years of happiness and great usefulness; therefore be it

*Resolved* 1. That we, the students of the Atlanta Medical College, extend hereby our condolence to our beloved dean and professor in this hour of greatest bereavement.

2. That we wish for him solace to his sorrowing heart and strength to his feeble frame in order that he may yet spend years of usefulness in the pursuit of that noble calling which he has so nobly filled.

3. That a copy of these resolutions be sent to Dr. Miller, published in the daily papers of the city, in THE ATLANTA MEDICAL AND SURGICAL JOURNAL and in the *Southern Medical Record.*

M. G. CAMPBELL, H. M. SMITH,
T. C. JEFFORDS, W. J. SHAW,
J. L. SANBORN, Committee.

---

### ELEVENTH INTERNATIONAL MEDICAL CONGRESS.

A letter directed to the undersigned by the Secretary-General of the Eleventh International Medical Congress and dated December 19th, 1893, contains the following communications:

"American members will pay on the English, French, and Italian railways single fares for double journeys, and will obtain a reduction of twenty per cent. on fares for Italian round-trip tickets.

"The documents required for their identification will be sent to you in January, and Americans intending to visit the Congress will have to apply to you for them.

"Full particulars concerning the journeys will accompany the documents.

"Messrs. Thos. Cook and Son, London, Paris, Rome and Naples, should be applied to for accommodation and for tickets for the excursion at Rome, Naples, and to Sicily. Such excursions will be arranged at Rome under the guidance of Mr. Forbes, member of several scientific societies and correspondent of the "*Times*"—for Naples, three days, including Vesuvius, Pompey, Capri, Sorrento, Castellamare, Baja, etc.—for Sicily, ten days from Naples, including Messina, Taormina, Catania, Girgenti, Siracusa, Palermo, and return to Naples.

The fares for members of the Congress will be considerably reduced and comprise hotel accommodations, carriages, guides, boats, etc.—about 70 frcs. each, for the three days, and 285 frcs. for the ten days.

"Full particulars concerning these excursions will be contained in a leaflet to be added to the instructions and documents for the journey."

From former communications the following are herewith quoted: The members' fee is five dollars, that of their wives or adult relations two dollars each. Checks or money orders may be sent to

Prof. L. Pagliani, Rome, Italy. Credentials have been promised in the near future. When they arrive (none were received last year), they may be too late for many who have started or are about to start. The undersigned, who is not informed of the cause of delay, proposes to supply in as official a form as he thinks he is justified in doing, credentials which are expected to be of some practical value. The North German Lloyd has promised to recognize them. It is suggested, besides, that a passport may increase the traveler's facilities.

Only the North German Lloyd (22 Bowling Green) and the Compagnie Générale Transatlantique (3 Bowling Green) have thought fit to grant any reductions to Congressists.

The reductions on Italian railways are available from March 1st to April 30th.

A. JACOBI, M. D.,

110 W. 34th Street, New York.

